# The role of theory of mind, group norms and intentionality in children's and adolescents' moral evaluations of a misinformer

**DOI:** 10.1111/bjdp.12544

**Published:** 2024-12-23

**Authors:** Aqsa Farooq, Anna Adlam, Adam Rutland

**Affiliations:** ^1^ University of Exeter Exeter UK; ^2^ Present address: University of Amsterdam Amsterdam The Netherlands

**Keywords:** adolescents, children, misinformation, moral evaluations, theory of mind

## Abstract

Misinformation poses a significant threat to modern society. Children and adolescents, highly active on social media, are particularly vulnerable to encountering misinformation from peers. Assessing whether intentionality impacts moral evaluations of misinformers, considering age and group norms, is crucial. Theory of Mind (ToM) plays a key role in understanding false beliefs and intentions. In a study involving 266 UK‐based children (8‐11‐years‐old) and adolescents (12‐15‐years‐old), participants evaluated a misinformer in a scenario involving a school competition. Deliberate misinformation led to harsher judgements and a higher likelihood of punishment. However, children tended to be more inclusive than adolescents regardless of intentionality. Adolescents with higher ToM believed in the misinformation less. Higher ToM correlated with harsher misinformer evaluations across the sample. These findings underscore the impact of intentionality, ToM and age on moral evaluations, suggesting that high ToM may mitigate positive feelings towards misinformers, potentially reducing misinformation acceptance.

**Statement of Contribution**

**What is already known on this subject?**

- Children negatively morally evaluate transgressions committed intentionally rather than unintentionally.
- Theory of Mind ability helps in understanding intentionality and reduces susceptibility to misinformation belief.
- Groups, and their norms, are crucial during childhood and adolescence.

**What does this study add?**

- Children and adolescents morally evaluate a deliberate misinformer more negatively than an accidental one.
- Higher Theory of Mind ability predicted harsher moral evaluations of the misinformer.
- Group norms did not influence moral evaluations of the misinformer.

In the contemporary digital landscape, social media platforms, notorious for hosting a plethora of inaccurate and unsubstantiated information, are frequently relied upon by children and adolescents for interaction with peers, social relationship formation and news consumption (Ofcom, 2020; Shin et al., 2018). This widespread circulation of *misinformation*, recognised globally as a significant threat to modern society (Lewandowsky et al., 2017), can come from various sources—including members of one's peer group. In fact, young people in particular are prone to sharing the unreliable information they come across online on the basis of their peership with the source, rather than the veracity of the content (Herrero‐Diz et al., 2020). It is therefore crucial to understand which factors contribute to children's and adolescents' evaluations of peer group members who share misinformation. Misinformation relies on its truth‐like appearance in order to circulate (Guess & Lyons, 2020) and is most persistent online, such as on social media (Shin et al., 2018; Vosoughi et al., 2018). The present research focuses primarily on children's and adolescents' moral evaluations of someone who shares misinformation in an online environment, as these evaluations can help determine what matters most to the individual, and what is likely to influence intentions and actions (Killen & Smetana, 2015).

When children and adolescents morally evaluate a moral transgressor, the intentionally of the transgressor can have a significant impact on their moral judgements (Wimmer et al., 1984). Moral transgressions, such as sharing falsehoods, receive harsher punishment when they are committed intentionally rather than unintentionally (D'Esterre et al., 2019). Similarly, sharing misinformation is one such act which can have contrasting moral implications depending on whether the sharing was accidental or deliberate. Being able to distinguish between intentionally and unintentionally spread misinformation is important for understanding whether someone is acting with malicious intent, for which appropriate sanctions may be necessary, and understanding whether someone is acting with negligence, in which case correction and new information may be sufficient (Zhou et al., 2022). For this reason, the current study explores the role of the misinformer's intentionality on children's and adolescents' moral evaluations of a misinformer. This exploration aims to extend past research which has focused on *children*'s moral evaluations of an individual either intentionally or unintentionally sharing falsehoods (D'Esterre et al., 2019). Given the importance of peer groups during childhood and adolescence, as well as the role of socio‐cognitive factors in understanding both intentionality and moral transgressions, the current study will also explore the role of group norms, and developmental differences in Theory of Mind, on children's and adolescents' moral evaluations of an accidental or deliberate misinformer. This investigation can help determine which factors make children and adolescents more or less likely to support those who spread misinformation.

Children's decisions about whether an individual's intentions were deliberate or accidental can rely on developmental factors such as their age and cognitive abilities (Wimmer et al., 1984). As demonstrated in past research (Farooq et al., 2022; Jambon & Smetana, 2013; Killen et al., 2011), with age, children's moral evaluations of ambiguous and accidental moral transgressors become more positive, as they become more forgiving of unintentionally committed transgressions. This developmental difference can be explained by the research that suggests that as children get older, their cognitive capacity also increases, allowing them to form complex, multifaceted evaluations about events that consider multiple perspectives (Nucci & Turiel, 2009; Wainryb et al., 2005). The skill that allows children to consider different perspectives is known as Theory of Mind (ToM; Lagattuta et al., 2015).

ToM is an important social and cognitive skill that allows individuals to ascertain others' intentions, emotions and beliefs in different social contexts (Premack & Woodruff, 1978). ToM is an ability that does more than help with inferring the intentionality of others; a growing body of research has brought attention to the association between children's ToM and their moral judgements (Baker et al., 2021; Lagattuta & Weller, 2013). For example, children who pass a ToM assessment without being misled tend to make more favourable moral evaluations of an unintentional transgressor compared to an intentional transgressor (D'Esterre et al., 2019). Thus, we decided to explore the role of both age and ToM in moral evaluations of the misinformer after their intentions were revealed as either accidental or deliberate.

Past research also suggests that ToM plays a key role in preventing children's false beliefs when presented with misleading information, though this research tends to be focused on early childhood (Bright‐Paul et al., 2008; Templeton & Wilcox, 2000). It is possible that for older children and adolescents, ToM ability is a predictor of resistance to false beliefs, particularly due to the literature suggesting that children's ToM competence helps with their understanding of false claims (Welch‐Ross, 1999). However, limited research explores the link between ToM and false beliefs, and the role age plays in this relationship, beyond middle childhood and into adolescence. Measuring ToM ability in the present study may therefore provide an important insight into the developmental factors that influence children's and adolescents' belief in misinformation, that measures of age alone cannot provide. Given the importance of ToM ability in preventing the formation of false beliefs and the lack of research exploring the link between ToM ability and misinformation belief beyond early childhood, we sought to fill this research gap. Based on measures used in previous research (Killen et al., 2011; Wellman & Liu, 2004) we asked participants to report how much they believed in the misinformation shared by their peer, in order to explore the relationship between ToM ability and misinformation belief amongst both children and adolescents.

During the developmental period from middle childhood to adolescence, it is also important to note that social groups play a crucial role in moral development. From early childhood, children become aware of differences between individual members of their own social group (ingroup) and members of a different social group (outgroup) (Aboud, 1993; Patterson & Bigler, 2006). Children aged six start to display strong ingroup bias for individuals who share group membership with them (Abrams, Rutland, & Cameron, 2003; Abrams, Rutland, Cameron, & Marques, 2003) and continue to do so through to adolescence (Raabe & Beelmann, 2011). Even children's information acceptance is subject to ingroup bias: positive information about ingroup members was accepted by 6‐7‐year‐olds regardless of the group membership of the source, but positive information about outgroup members was only accepted from an ingroup source (Aldan & Soley, 2019). Hence, children tend to be more preoccupied with group membership factors than adolescents, preferring their ingroup members due to their ingroup status above other factors such as their behaviour (Abrams et al., 2008; Abrams, Rutland, & Cameron, 2003; Abrams, Rutland, Cameron, & Marques, 2003).

An important tenet of group membership involves following the norm of the ingroup, as the norm encompasses the core values unique to the group, indicating the beliefs and behaviours group members should align themselves with (Nipedal et al., 2010). The norm of the group is influential to children's moral evaluations (Hitti et al., 2014), and can even encourage children to have more positive and empathetic attitudes towards outgroup peers (Nesdale et al., 2005) despite their loyalty to the ingroup (Abrams et al., 2008). Loyalty to the ingroup is a widely upheld norm of group membership (Levine & Moreland, 2002), but it can also play a role in the spread of misinformation. For instance, loyalty to the ingroup often directly contends with being objective in seeking the truth, resulting in the continued spread of misinformation (Van Bavel & Pereira, 2018). This is because being loyal may require believing a source of information due to its group affiliation, rather than the truthfulness of the information—something we know adolescents tend to do (Herrero‐Diz et al., 2020). It is therefore important to understand whether these conflicting values, if encapsulated as group norms, may influence moral evaluations of the peer group members who misinform. To explore the role of the participants' group's norm on moral evaluations of a misinformer from the ingroup, we manipulated the group norm in the present study so that participants were either exposed to a group norm that emphasized ingroup loyalty, or a group norm that prioritized truth‐seeking.

The present study aimed to explore how the group norm (ingroup loyalty vs. truth‐seeking), the misinformer's intentionality (accidental vs. deliberate) and ToM ability influence children's and adolescents' moral evaluations of a misinformer. We also explored the role of age and ToM ability on participants' belief in the misinformation shared by their ingroup peer. Congruent with past research exploring developmental trends in relation to group dynamics, such as group membership and group norms (Abrams, Rutland, & Cameron, 2003; Abrams, Rutland, Cameron, & Marques, 2003; Farooq et al., 2022; Hitti et al., 2014; Nesdale et al., 2005), the current study simulated a context where participants were asked to identify with their (hypothetical) ingroup.

We asked participants whether they believed the misinformation shared by their ingroup peer about their outgroup peer. Due to the literature suggesting that children's ToM competence helps with their understanding of false claims (Welch‐Ross, 1999), we expected participants to be less likely to believe the misinformation as their ToM ability increased, but we expected this positive relationship to rely on the age of the participant. Thus, we expected age to moderate the relationship between participants' ToM and belief in misinformation. For this exploration, it was necessary to compare children to adolescents developmentally, as categorical groups based on the UK school system, with the children in sample being primary school pupils and the adolescents of the sample being secondary school pupils.

We also asked participants to provide their moral evaluations of the misinformer. This involved providing a judgement of the misinformer, deciding whether or not to include the misinformer and assigning punishment to the misinformer. For the participants who were told that the ingroup peer had misinformed deliberately, we expected harsher moral evaluations compared to those who were told that their ingroup peer had misinformed accidentally. This is based on research with children which showed that individuals who intentionally shared a false claim were assigned harsher punishments compared to the individuals who did so unintentionally (D'Esterre et al., 2019). The same research also suggests that passing ToM assessment tasks is associated with judging an unintentional transgressor more favourably than an intentional transgressor. Hence, we also expected that the participants with higher ToM ability will be more likely to negatively morally evaluate the deliberate misinformer relative to the accidental misinformer.

We also expected harsher moral evaluations of the misinformer to be attributed by participants assigned to the group with a truth‐seeking norm compared to those assigned to a group with a loyalty norm. This was based on past research demonstrating that children and adolescents pay close attention to the norm of their group when making moral evaluations, as well as research suggesting that loyal ingroup peers are liked, and loyalty to the ingroup is regarded as an important way of maintaining group membership (Abrams & Rutland, 2012; Killen et al., 2013; Rutland et al., 2015). Thus, a misinforming ingroup peer would be favoured more when their group norm emphasizes being loyal to fellow ingroup peers, and comparatively a misinforming ingroup peer would be favoured less when their group norm emphasizes truth‐seeking.

## HYPOTHESES

The positive relationship between participants' ToM ability and their belief in the misinformation would be moderated by their age.

Participants will make more positive moral evaluations about an ingroup peer who accidentally misinformed compared to an ingroup peer who deliberately misinformed.

Participants assigned to the group with a loyalty norm would make more positive moral evaluations about the ingroup misinformer compared to those assigned to the group with a truth‐seeking norm.

Participants with higher ToM ability will be more likely to negatively morally evaluate the deliberate misinformer compared with the accidental misinformer.

## METHOD

### Participants

Participants from a primary and secondary school in South‐West England were recruited for this study. After the removal of the 27 participants who failed the manipulations checks (20 were children), a total of 266 participants made up the sample. There were 133 children (68 girls, 65 boys) aged 8–11 ($M_{\mathrm{age}}$ = 9.33, *SD* = .94) and 133 adolescents (66 girls, 62 boys, 5 non‐binary) aged 12–15 ($M_{\mathrm{age}}$= 13.5, *SD* = .93). The sample had an ethnic breakdown of 62% White British, 14% other (e.g. Eastern European), 8% Asian British (Chinese, Indian, Bengali, Pakistani), 2% Mixed Race/Dual Heritage and 2% Black British, while 17% did not answer. This sample size was determined using G*Power by conducting an a priori power analysis for linear regression analyses with multiple predictors using an alpha of .05, a power of .90 and a small‐medium effect size (*η* ^*2*^ = .15) (Faul et al., 2007). This calculation estimated a required sample size of 190. Parental consent was obtained for all participants.

### Design

This study used a 2 (age group: children vs. adolescents) × 2 (group norm: ingroup loyalty vs. truth seeking) × 2 (misinformer intentionality: accidental vs. deliberate) between‐participants design.

### Procedure

Participants were told about a National Spelling Bee Competition. To establish group membership with their school, participants chose a logo and a mascot for their school team. Then, they were told more about the competition, which was happening between their school and other schools across the country, for which the winning school receives a trophy. There is one strict ‘Golden Rule’ which all teams must follow: ‘Do not turn up late’. Breaking this rule can result in points deduction. Participants were then told that their school has successfully reached the final of the competition which is against The Willow Tree School, with the final game determining who gets to win the competition.

See Table 1 for the full details of this procedure and the manipulations. Participants were first randomly assigned to one of the two group norm conditions (1) which they were initiated to with a message (2). Then, they saw a comment from the misinformer in the WhatsApp group chat for the competition, accusing an outgroup competitor of breaking the Golden Rule (3). This was followed by the group norm‐congruent reaction of their three fellow ingroup peers (4). Finally, they were randomly assigned to the misinformer intentionality condition (5) revealing what the misinformer knew *before* sharing the false claim (6). The study measures followed (7).

**TABLE 1:** Study procedure indicating the order and content of the study manipulations, followed by the measures.

(1) Group norm manipulation	Ingroup loyalty	Truth‐seeking
(2) Group norm message	Welcome to the team. Our goal is to win this competition! Now that you are a member of this team, you should know what is important to us. We value loyalty. We think that we should always believe our group members and support them no matter what.	Welcome to the team. Our goal is to win this competition! Now that you are a member of this team, you should know what is important to us. We value seeking the truth. We think that we should make sure something is true before we believe it, no matter who it comes from.
(3) Comment from the misinformer (Sam) about outgroup competitor (Alex)	Alex was late to the final! Alex broke the Golden Rule!	
(4) Ingroup peers' reactions	I believe Sam no matter what. Sam is 100% right	Not 100% sure if this is true, going to wait for more information
(5) Misinformer intentionality manipulation	Accidental	Deliberate
(6) Revelation of misinformer's intentions	BUT… Sam did not know that the time of the competition final has been changed by the judges, so Alex was not late and had not broken the Golden Rule! Sam had not checked this information before posting the video and making the comment on WhatsApp	BUT… Sam knew that the time of the competition final has been changed by the judges, which meant that she also knew that Alex was not late and had not broken the Golden Rule! Sam still decided to post the video and make the comment on WhatsApp
(7) Study measures	Belief in misinformation Moral evaluations (Judgement, inclusion, punishment) of the misinformer Theory of mind	

*Note*: Participants were randomly assigned to the group norm conditions and the misinformer intentionality conditions. The characters in the procedure were always gender‐matched to the participants.

Group norm manipulation checks were carried out twice during the procedure before participants were shown the measures. Participants were asked, ‘What does your school group value the most?’ Answers were multiple choice: 1 (‘Being funny, which means always trying to have fun), 2 (‘Being loyal, which means trusting and supporting teammates no matter what’), 3 (‘Being truth‐seeking, which means finding out the truth no matter what’). Participants who failed this manipulation check were excluded from the final analyses.

### Measures

#### Belief in misinformation

‘Do you believe that Alex, from the Willow Tree School, broke the Golden Rule of the competition?’ Participants answered on a 5‐point Likert scale ranging from 1 (*Definitely not*) to 5 (*Definitely yes*).

#### Judgement of misinformer

‘Sam, from your school's team, posted the video to WhatsApp. How do you feel about Sam?’ Participants selected their response from a 5‐point scale showing faces ranging from 1 (very unhappy face) to 5 (very happy face).

#### Inclusion of Misinformer

‘Do you want Sam to still be in your team?’ Participants selected their answer from a 5‐point Likert scale which went from 1 (*Definitely not*) to 5 (*Definitely yes*).

#### Punishment of misinformer

‘Do you think Sam should be punished for his/her actions?’ Participants selected their answer from a 5‐point Likert scale which went from 1 (*Definitely not*) to 5 (*Definitely yes*).

#### Theory of mind (ToM)

Participants' ToM was measured using the Strange Stories questions (Happé, 1994; White et al., 2009). This is one of the most common ways of measuring advanced ToM ability, as demonstrated by their use in research with older children, adolescents and young adults (Gönültaş et al., 2020; Perez‐Zapata et al., 2016). Of the numerous Strange Stories, the four interpersonal stories (double bluff, persuasion, white lie and misunderstanding) were utilized for the present study, as they specifically required children and adolescents to understand and interpret the intentions, beliefs and emotions of others. Participants read the four scenarios, and at the end of each, they were asked a question which required them to understand the mental states of the gender‐matched story characters (see Table 2). These answers were coded by two research assistants independently, accordingly: 2 = correct answer, where mental state is accurately attributed; 1 = correct information is given, but without attributing mental states and 0 = incorrect and/or irrelevant information. Interrater reliability was assessed based on 25% of responses, and there was a high level of agreement (Cohen's *κ* = .90). Participants were then given a total ToM score out of eight, based on their responses to the four stories. Those participants who answered less than three of the questions could not be given a ToM score (*n* = 48), and those who gave three answers were given a fourth via the multiple imputation method (*n* = 38) (Wayman, 2003). The final number of participants (*n* = 218) was still sufficient for attaining the desired amount of power for the analyses we had planned to conduct.

**TABLE 2:** Strange stories (White et al., 2009) used to measure ToM ability.

Strange story	Scenario	Question
Double bluff	Simon is a big liar. Simon's brother Jim knows this, he knows that Simon never tells the truth! Now yesterday Simon stole Jim's ping‐pong paddle, and Jim knows Simon has hidden it somewhere, though he can't find it. He's very cross. So he finds Simon and he says, ‘Where is my ping‐pong paddle? You must have hidden it either in the cupboard or under your bed, because I've looked everywhere else. Where is it, in the cupboard or under your bed’? Simon tells him the paddle is under his bed.	Why will Jim look in the cupboard for the paddle?
Persuasion	Brian is always hungry. Today at school it is his favourite meal—sausages and beans. He is a very greedy boy, and he would like to have more sausages than anybody else, even though his mother will have made him a lovely meal when he gets home! But everyone is allowed two sausages and no more. When it is Brian's turn to be served, he says, ‘Oh, please can I have four sausages, because I won't be having any dinner when I get home!’	Why does Brian say this?
White Lie	Harry waited all year for Christmas, because he knew at Christmas he could ask his parents for a rabbit. Harry wanted a rabbit more than anything in the world. At last Christmas Day arrived, and Harry ran to unwrap the big box his parents had given him. He felt sure it would contain a little rabbit in a cage. But when he opened it, with all the family standing round, he found his present was just a boring old set of encyclopedias, which Harry did not want at all! Still, when Harry's parents asked him how he liked his Christmas present, he said, ‘It's lovely, thank you. It's just what I wanted.’	Why did he say this?
Misunderstanding	A burglar who has just robbed a shop is making his getaway. As he is running home, a policeman on his beat sees him drop his glove. He doesn't know the man is a burglar, he just wants to tell him he dropped his glove. But when the policeman shouts out to the burglar, ‘Hey, you! Stop!,’ the burglar turns round, sees the policeman and gives himself up. He puts his hands up and admits that he did the break‐in at the local shop.	Why did the burglar do that?

#### Control measures

We measured participants' spelling interest (‘how much do you like spelling?’ answered on a 5‐point Likert scale) and identification with their school group (‘how much do you like being a part of your school's team?’ answered on a 5‐point Likert scale). These measures were added as control variables in each of the following analyses. We conducted randomisation tests to ensure participants' gender, spelling interest and school group identification was balanced across the age groups and experimental conditions. Chi‐square tests showed that gender (looking only at those who identified as either boy or girl, as the non‐binary identifying participants were exclusively adolescents) did not differ significantly across the age groups, χ2 (1, *N* = 261) 147 = .005, *p* = .944, group norm conditions, χ2 (1, *N* = 261) = .353, *p* = .552, or misinformer intentionality conditions, χ2 (1, *N* = 261) = .004, *p* = .949. Independent t‐tests showed that there were no age related differences in spelling interest *t*(226) = 1.46, *p* = .073, however, children (*M* = 3.92, *SD* = 1.11) liked being in their school group more than adolescents did (*M* = 3.29, *SD* = 1.18), *t*(226) = 4.06, *p* < .001. Across the group norm conditions, participants did not significantly differ by their interest in spelling, *t*(226) = −.004, *p* = .498, nor their identification with their school group, *t*(226) = .202, *p* = .420. Across the misinformer intentionality conditions, participants also did not significantly differ by their interest in spelling, *t*(226) = −.411, *p* = .341, nor their identification with their school group, *t*(226) = .413, *p* = .340. As we asked participants the school identification question at the very end of the survey, and still found no differences according to group norm, suggests that participants in both conditions equally identified with their school group. The mean scores for school identification in each group norm condition (ingroup loyalty *M* = 3.67, *SD* = 1.22; truth‐seeking *M* = 3.64, *SD* = 1.12) were also above the midpoint of the 5‐point scale, suggesting an overall propensity to favour being part of their school group.

To explore H1, a moderation analysis was conducted using the PROCESS macro in SPSS, Model 1 (Hayes, 2018), where ToM was entered as the independent variable, Belief in Misinformation was the dependent variable and age was the dichotomous moderator variable.

To explore H2 and H3 concerning participants' judgement, inclusion and punishment evaluations of the misinformer, three linear regression analyses were conducted for each measure. In each regression analysis, the following were added as predictors: age group (0 = children, 1 = adolescents), group norm (0 = ingroup loyalty, 1 = truth‐seeking), misinformer intentionality (0 = accidental, 1 = deliberate) and ToM score. All of the interaction terms between variables were also added to a separate model, however the inclusion of the interaction terms did not significantly account for the variance in the outcome interest in the overall model, resulting in their exclusion from the final models, and the rejection of H4.

Correlations between all study variables were explored with bivariate correlation analyses (Table 3).

**TABLE 3:** Partial correlations between study variables.

	1	2	3	4	5	6	7	8
1. Age group	1							
2. Theory of mind	.18**	1						
3. Group norm	.02	.04	1					
4. Misinformer intentionality	−.02	.04	−.02	1				
5. Belief in misinformation	−.17**	−.25**	−.06	.03	1			
6. Judgement of misinformer	.01	−.22*	.20**	−.16**	.12	1		
7. Inclusion of misinformer	−.19**	−.06	−.01	−.10	.02	.29**	1	
8. Punishment of misinformer	−.09	.12	−.15*	.23**	−.16**	−.55**	−.29**	1

*Note*: Age group (0, children; 1, adolescents); group norm (0, ingroup loyalty; 1, truth‐seeking); misinformer intentionality (0, accidental; 1, deliberate).

*p < .05, **p < .01 (2‐tailed).

## RESULTS

The moderation analysis revealed that the relationship between ToM and belief in misinformation is moderated by age, (*b* = −.25, 95%, CI [−.46, −.04] *t* = −2.36, *p* = .019) supporting H1.

**FIGURE 1:**
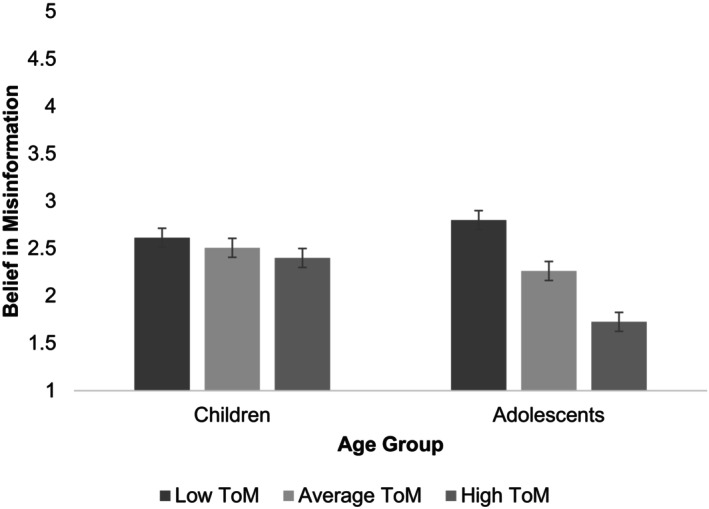
Participants' belief in misinformation (‘Do you believe that Alex, from the Willow Tree School, broke the Golden Rule of the competition?’ 1, definitely not; 5, definitely yes) by age group and ToM ability (with standard error bars +/− .1). Overall, 39% selected ‘Definitely not’; 11% selected ‘Probably not’; 28% selected ‘I'm not sure’; 17% selected ‘Probably yes’; 5% selected ‘Definitely yes’.

Simple slopes analysis showed that for the children of the sample, there was a non‐significant relationship between ToM and belief in misinformation, *b* = −.06, 95%, CI [−.20, .09], *t* − .88, *p* = .382. For the adolescents of the sample, there was a significant negative relationship between their ToM and belief in misinformation, *b* = −.31, 95%, CI [−.47, −.16] *t* = −3.96, *p* < .001. Taken altogether, this analysis shows that amongst adolescents, higher ToM ability means less belief in misinformation (see Figure 1).

A multiple linear regression analysis showed that the model with all variables added as predictors (age group, group norm, misinformer intentionality and ToM) was significant, *F* (4, 216) = 5.63, *p* < .001, explaining 10% (*R* ^2^ = .10) of the variance in participants' Judgement of the Misinformer evaluation (Table 4). As predicted in H2, misinformer intentionality was a significant predictor of participants' judgements of the misinformer (*p* = .011). Participants who read about an accidental misinformer judged the misinformer more positively compared to the participants who read about a deliberate misinformer. ToM was also a significant predictor of participants' judgements of the misinformer (*p* < .001). Participants with higher ToM were more likely to negatively judge the misinformer compared to participants with lower ToM. The group norm was not a significant predictor, contrary to H3.

**TABLE 4:** Linear regression analyses for the moral evaluations of the misinformer.

Predictors	Judgement of misinformer			Inclusion of misinformer			Punishment of misinformer		
	*B*	SE	*β*	*B*	SE	*β*	*B*	SE	*β*
Age group	.17	.13	.09	−.50	.16	−.20*	−.28	.15	−.13
Group norm	.22	.13	.12	.18	.16	.07	−.08	.15	−.04
Misinformer intentionality	−.32	.12	−.17*	−.24	.16	−.10	.55	.14	.25**
ToM	−.13	.04	−.23**	−.01	.05	−.02	.08	.04	.13
Adjusted *R* ^2^	.10			.06			.10		

*p < .05, **p < .001.

For participants' inclusion of the misinformer, the model with all variables added as predictors was significant, χ^2^ (4, 212) = 20.68, *p* < .001 (Table 4). However, only age group was a significant predictor of participants' inclusion of the misinformer (*p* = .003); children favoured inclusion of the misinformer more than adolescents. As none of the other variables in the model were significant, H2 and H3 were not supported.

For participants' punishment of the misinformer, the model with all variables as predictors was significant χ^2^ (4, 209) = 25.20, *p* < .001 (Table 4). As per H2, misinformer intentionality was a significant predictor of participants' punishment of the misinformer (*p* < .001). The participants who read about a deliberate misinformer were more likely to support punishment of the misinformer compared with the participants who read about an accidental misinformer. Furthermore, ToM's significance as a predictor of participants' punishment evaluations of the misinformer was just above the threshold of significance (*p* = .053); suggesting a weak trend towards participants with high ToM being more likely to agree with punishing the misinformer compared to participants with low ToM. H3 was not supported, as participants' evaluations did not differ by their group's norm. See Table 5 for means and standard deviations as a function of categorical predictors.

**TABLE 5:** Proportions of participants' moral evaluations of the misinformer as a function of categorical predictors.

	Judgement of misinformer^a^		Inclusion of misinformer^b^		Punishment of misinformer^c^	
	M	SD	M	SD	M	SD
Age group						
Children (*n* = 133)	2.32	1.07	3.12	1.26	3.62	1.18
Adolescents (*n* = 133)	2.34	.88	2.65	1.15	3.43	1.02
Group norm						
Ingroup loyalty (*n* = 143)	2.30	1.01	2.88	1.22	3.53	1.11
Truth‐seeking (*n* = 123)	2.36	.94	2.89	1.24	3.52	1.10
Misinformer intentionality						
Accidental (*n* = 134)	2.48	.94	3.01	1.24	3.28	1.12
Deliberate (*n* = 132)	2.17	.99	2.76	1.21	3.78	1.03

Abbreviations: M, mean proportions; *n*, number of participants; SD, standard deviations.

^a^ ‘Sam, from your school's team, posted the video to WhatsApp. How do you feel about Sam?’ (1 = very unhappy face; 5 = very happy face).

^b^ ‘Do you want Sam to still be in your team?’ (1, definitely not; 5, definitely yes).

^c^ ‘Do you think Sam should be punished for his/her actions?’ (1, definitely not; 5, definitely yes).

## DISCUSSION

This study extends previous research exploring children's moral evaluations of intentional and unintentional transgressors by investigating how age, ToM ability and group norms influence children's and adolescents' moral evaluations about deliberate and accidental misinformers. Our results show that participants who read about an ingroup peer who misinformed deliberately attributed harsher judgements and punishment evaluations, than those who read about an ingroup peer who misinformed accidentally. Participants' age and ToM ability also played a role in these evaluations. Children were more likely to include the misinformer compared to adolescents, and higher ToM ability predicted harsher moral evaluations of the misinformer—regardless of intentionality. Amongst adolescents, higher ToM ability was associated with less belief in misinformation. The norm manipulation did not have any influence on moral evaluations.

These findings suggest that, as expected, the intentionality of a misinformer is key to how they are judged and assigned punishment by participants, with deliberately misinforming regarded as morally worse than accidentally misinforming. This supports previous research indicating that children assign more punishment to an intentional transgressor than an unintentional transgressor (D'Esterre et al., 2019), and extends it by showing this trend to continue until adolescence. This suggests that children and adolescents recognize the importance of intentionality when committing a transgression, and evaluate the transgressor accordingly—even when they are from within the ingroup. Distinguishing between those who act with deceitful intentions and those who act neglectfully is important in the age of misinformation prevalence, as it can help identify those individuals who may have malicious objectives, investigate their motives, and thus prevent further ill‐intentioned actions that disadvantage others (Zhou et al., 2022). Information about intentionality is not always available, as it was in the present study. However, this study suggests when children and adolescents are confronted with misinformation, parents, teachers and facilitators should encourage them to *consider* and *question* the intentionality and possible motives of the misinformer, as a way of getting them to think about intentionality when the information is not available.

The present study also extends past research by demonstrating participants' preference for an unintentional (accidental) over intentional (deliberate) transgressor in a competitive intergroup context, where there is arguably an advantage for the participants' ingroup as a result of the misinformer's claim. However, it is perhaps the competitive intergroup context which results in a lack of difference in participants' inclusion evaluations of the deliberate or accidental misinformer. It is known from past research that competitive intergroup contexts elicit strong preferences for ingroup peers who behave in a way that results in an advantage for the group, even if their behaviour is counter‐normative (McGuire et al., 2019). This suggests that even though the participants from the present study were more willing to negatively judge and recommend punishment for the deliberate misinformer, they likely did not regard this as a reason to *not* include the misinformer compared with those who read about an accidental misinformer—particularly with the competition still in progress, and the competitive disadvantage of losing a team member. Although, the age‐related differences in the results may shed light on this further.

In the present study, we found that children favoured inclusion of the misinformer more than adolescents, regardless of the misinformer's intentionality. This finding can be explained by past research, which shows that children like their (loyal) ingroup peers, and when deciding whether or not to exclude them, their ingroup status can take precedence over other considerations such as the peer's behaviour, the group's norm, etc. (Abrams et al., 2008; Abrams, Rutland, & Cameron, 2003; Abrams, Rutland, Cameron, & Marques, 2003). These other considerations become more important later on, in adolescence, when the ingroup status of a peer no longer becomes the sole reason to include a peer group member. This can be explained by social domain theory (SDT) research, which shows that children give precedence to moral concerns, such as harm and fairness, and adolescents to group‐related matters when providing justifications for their moral evaluations of moral transgressors (Killen et al., 2013; Killen & Rutland, 2011; Rutland et al., 2015). Hence, it is possible that the driving concern in children's inclusion of the misinformer is related to the moral domain, such as the unfairness of leaving out the misinformer, a member of the ingroup, from the group. It is therefore crucial to investigate further with empirical research, whether children's propensity to be more fairness‐minded in inclusion decisions also makes children more susceptible to believing and sharing misinformation from ingroup peers. The present study's measure of misinformation belief and the role of ToM provides further insight on the age‐related differences in evaluations of the misinformer.

This study found that higher ToM ability was associated with less belief in misinformation, as was expected based on previous studies (Smetana et al., 2013; Welch‐Ross, 1999); however, we found this only to be the case amongst adolescents. Given that previous work has only focused on children in early childhood, the present study's finding provides a novel insight by showing that misinformation effects can be prevalent even beyond childhood, and that ToM ability continuously plays a role throughout those developmental years. This has significant implications for the spread of misinformation, as it suggests that despite being told that a claim is definitely false, having low ToM ability can hinder even adolescents' ability to shed their false belief. When encountering false information online, those adolescents with low ToM may be most susceptible to developing false beliefs, even after being presented with correcting information with the facts. It is possible that having higher ToM leads to a scepticism towards the misinformer's claim, hence why participants with higher ToM were also more likely to negatively judge and recommend punishment for the misinformer. Those with lower ToM may have considered other factors in their decisions about including the misinformer, which may be linked to their increased susceptibility to the false claim and less harsh moral evaluations of the misinformer, regardless of their intentionality. Hence, to tackle the spread of misinformation, it is important for teachers, parents and facilitators to recognize the role of age and ToM ability as factors that can make children and adolescents vulnerable to misinformation belief. It is also important to further investigate why children and those with lower ToM ability are more likely to favour a misinformer, in order to better understand the reasons that underlie their vulnerability to misinformation. However, it should be noted that overall, participants' belief in the misinformation was relatively low compared to the midpoint of the scale, suggesting that participants were largely sceptical—likely as a result of being told that the information was false. For future research, it would be important to delve deeper and further underpin the factors that may be responsible for why individuals may hold incorrect beliefs known to be false, particularly as we know how such phenomena contributes to the continued spread of partisan misinformation in the adult world (Del Vicario et al., 2017; Lees & Cikara, 2020; Van Bavel & Pereira, 2018).

Finally, we did not find effects of the group norm manipulation. Participants did not morally evaluate the misinformer any differently when their group norm emphasized ingroup loyalty or truth‐seeking. This could have been due to the competitive nature of the study's context, resulting in the participants assigned to the truth‐seeking peer group norm being reluctant to negatively judge the ingroup misinformer. Previous research with children suggests that when loyalty contends with moral concerns about principles of fairness or rightness, children will whistleblow on ingroup and outgroup peers equally when the moral transgression is mild—but when the moral transgression is *severe*, children were more reluctant to expose their ingroup members relative to outgroup members (Misch et al., 2018). It is therefore possible that amongst children *and* adolescents, the trade‐off between loyalty to the group and moral concerns of being truthful extends to the problem of misinformation being spread by ingroup members. In all conditions, the misinformer shared a false claim that accused an outgroup peer of breaking a low‐stakes competition rule. This was to prevent participants from getting distracted, and focus instead on the misinformer's actions. However, the lack of severity in the misinformers' claim may also have contributed to the lack of group‐norm effects. Though omitted from this paper, participants were also invited to provide open‐ended justifications for their moral evaluations. Analyses of these responses showed that participants with a truth‐seeking norm were more likely to reference lying (providing responses like, ‘because she's a liar’) when justifying their decisions about including the misinformer, compared with participants with a loyalty group norm. This suggests an influence of the group norm on participants' thought processes *behind* their moral evaluations, rather than their moral evaluations themselves, though further research would be needed to confirm this. Nonetheless, for children and adolescents to value truth‐seeking above ingroup loyalty in the context of misinformation, the current study suggests that peer‐group norms may be insufficient in curtailing the persuasive force of ingroup preferences in competitive intergroup contexts. Further research is warranted to explore whether this dynamic is limited to competitive contexts, or whether it is more pervasive, and also whether the severity of the accusation makes a difference.

In sum, this study extends previous work by exploring the role of age, ToM ability and group norms on both children's and adolescents' moral evaluations about an accidental or deliberate misinformer. Having better ToM ability did correlate with being less likely to believe the misinformation amongst adolescents, and across both age groups, higher ToM was associated with more negative moral evaluations of the misinformer. A misinformer with intentions to deliberately misinform was judged and punished more harshly by participants than a misinformer who accidentally misinformed. However, children were in favour of including the misinformer more than adolescents *regardless* of their intentionality, perhaps due to the ingroup status of the misinformer. The group norm manipulation did not have direct effects on participants' moral evaluations, suggesting countermeasures for misinformation amongst young people need to be focused more on individual‐level factors, such as age and ToM ability, than group‐level factors, such as group norms. Altogether, this study suggests that to tackle the spread of misinformation, it is important to understand whether children and adolescents consider the misinformer's intentions, which is key to their moral evaluations. The ability to do so, however, may rely on factors such as their ToM ability, which need to be further investigated amongst children and adolescents, due to the way they influence belief in misinformation.

## AUTHOR CONTRIBUTIONS

**Aqsa Farooq:** Conceptualization; investigation; formal analysis; writing – original draft; project administration. **Anna Adlam:** Conceptualization; writing – review and editing; supervision. **Adam Rutland:** Conceptualization; methodology; supervision; writing – review and editing.

## Data Availability

The data that support the findings of this study are available from the corresponding author upon reasonable request.
